# Ophthalmologic evaluation of severely obese patients undergoing bariatric surgery: A pilot, monocentric, prospective, open-label study

**DOI:** 10.1371/journal.pone.0216351

**Published:** 2019-05-16

**Authors:** Chiara Posarelli, Guido Salvetti, Paolo Piaggi, Francesca Guido, Giovanni Ceccarini, Ferruccio Santini, Michele Figus

**Affiliations:** 1 Ophthalmology, Department of Surgical, Medical, Molecular Pathology and of Critical Area, University Hospital of Pisa, Pisa, Italy; 2 Obesity Center, at the Endocrine Unit, University Hospital of Pisa, Pisa, Italy; 3 National Institute of Diabetes and Digestive and Kidney Diseases, National Institutes of Health, Phoenix, AZ, United States; Weill Cornell Medicine-Qatar, QATAR

## Abstract

**Purpose:**

The aim of this study was to investigate the pathogenic role of obesity on blinding eye diseases in a population of severely obese patients with no history of eye diseases, and to verify whether weight loss induced by bariatric surgery may have a protective effect.

**Methods:**

This was a pilot, monocentric, prospective, and open label study conducted at the University Hospital of Pisa. Fifty-seven severely obese patients with a mean body mass index value of 44.1 ± 6 kg/m^2^ were consecutively recruited and received a complete ophthalmological evaluation and optical coherence tomography. Twenty-nine patients who underwent gastric bypass were evaluated also 3 months, and 1 year after surgery.

**Results:**

At baseline, blood pressure value were directly and significantly related to intraocular pressure values (p<0.05, R = 0.35). Blood pressure values were also significantly and inversely related to retinal nerve fiber layer thickness, particularly in the temporal sector (RE p<0.05 r-0.30; LE p<0.01, R = -0.43). Moreover, minimum foveal thickness values were significantly and inversely associated with body mass index (RE p<0.02, R = -0.40; LE p<0.02, R = -0.30). A significant reduction of body mass index (p<0.05) and a significant (p<0.05) improvement of blood pressure was observed three months and one year after gastric bypass, which were significantly associated with an increase in retinal nerve fiber layer thickness and minimum foveal thickness values in both eyes (p<0.05).

**Conclusions:**

The results of this study suggest that obese patients may have a greater susceptibility to develop glaucomatous optic nerve head damage and age-related macular degeneration. Moreover, weight reduction and improvement of comorbidities obtained by bariatric surgery may be effective in preventing eye disease development by improving retinal nerve fiber layer and foveal thickness.

## Introduction

Obesity is a major public health problem, due to its increasing prevalence in most countries and the related health risks. Worldwide, the proportion of adults with a body-mass index (BMI) of 25 kg/m^2^ or higher is calculated to be greater than 35% both in men and in women [1].

Obesity and obesity-related systemic diseases determine a greater risk for blinding eye diseases, such as cataract, glaucoma and age-related macular degeneration (AMD) [2]. Bariatric surgery is currently the most effective mean to reach a substantial and stable body weight reduction in severe morbid obesity, with an important amelioration of various comorbidities [3]. The potential role of bariatric surgery in prevention and improvement of diabetic retinopathy [4], and its role in solving papillary oedema in intracranial hypertension has been recently investigated [5]. The association between vitamin deficiencies after bariatric surgery and related eye diseases is also well established [6]. Little is known about the effects of bariatric surgery on eye diseases of different aetiologies. In this study we measured several ophthalmologic parameters in a population of severely obese patients with no history of eye diseases, to get further insights into the pathogenic role of obesity on blinding eye diseases and to verify whether weight loss induced by bariatric surgery may have a protective effect.

## Subjects and methods

This was an explorative, prospective, monocentric open-label study. The study was conducted according to the principles defined in the Declaration of Helsinki and amendments, and was approved by the local Ethical Committee (Area Vasta Nord Ovest Ethical Committee–CEAVNO). Between July 2015 and January 2016, 57 consecutive obese patients, aged 18 to 65 years (mean age 46,2 ± 10), were selected among those attending the Obesity Center of the Endocrinology Unit, at the University Hospital of Pisa, Italy. All patients gave their written informed consent.

The inclusion criteria were BMI≥40 Kg/m^2^ orBMI≥35 Kg/m^2^ with co-morbidities. Patients with a diagnosis of type 2 diabetes or with pre-existing ocular diseases or visual alterations were excluded to avoid confounding factors related to pre-existing retinal pathology, independent from obesity *per se*. Patients with dementia, Parkinson’s disease, Alzheimer’s disease, multiple sclerosis or other severe neurological diseases were also excluded since ophthalmologic parameters may be altered in these conditions. Patients with lens opacities that made impossible the posterior segment examination, or with a refractive error > +5 or <-8 diopters spherical equivalent were also excluded.

The following parameters were recorded: age, body mass index (BMI), waist and hip circumference, heart rate, systolic blood pressure (SBP) and diastolic blood pressure (DBP), reported as average of two measurements in the sitting position with an interval of 2 minutes with an appropriate cuff. Blood glucose, glycated hemoglobin, fasting insulin, creatinine, fasting leptin, vitamin D, vitamin B, folic acid (Table 1), co-morbidities and concurrent diseases were also registered.

**Table 1:** Biochemistry tested before bariatric surgery.

Parameter	Mean	Std. Deviation	Range
Glucose mg/dL	102,6	33,2	69–288
HbA1c mmol/M	43,3	10,6	29–87
Fasting Insulin μU/L	19,1	14,6	10–53
Creatinine mg/dL	0,7	0,8	0,5–1,7
Fasting Leptin ng/mL	43,3	16,9	16–83
Vitamin D ng/mL	19,5	7,7	13–15
Vitamin B12 pg/mL	422,9	149,1	221–1008
Folic Acid ng/mL	6,8	2,6	4–16
Total Cholesterol mg/dL	191,8	35,6	114–257
LDL Cholesterol mg/dL	129,0	32,0	57–196
HDL Cholesterol mg/dL	48,3	10,9	28–72
Triglycerides mg/dL	113,7	44,2	41–254

HbA1c (glycated haemoglobin), LDL (low-density lipoprotein), HDL (high-density lipoprotein).

Twenty-nine patients underwent gastric bypass surgery (GBP) [7]. After surgery all patients received adequate daily vitamin supplementation, including 800 micrograms of Vitamin A. Anthropometric and ophthalmologic data were collected before, 3 months and one year after GBP.

### Ophthalmologic evaluation

In each eye, right eye (RE) and left eye (LE), the following data were collected: Visual acuity (Snellen equivalent); intraocular pressure (IOP); central corneal thickness (CCT); foveal thickness (FT) average value; FT minimum value; FT maximum value; average retinal nerve fiber layer (RNFL) thickness value; RNFL thickness superior, temporal, inferior, and nasal sector; cataract present or absent.

The anterior segment was examined by slit-lamp biomicroscopy, best corrected visual acuity was recorded as Snellen equivalent, opacity of the lens was classified using the Lens Opacities Classification System III (LOCS), and intraocular pressure was measured with Goldmann applanation tonometry. The posterior segment was examined after pupil dilation; central corneal thickness was measured with Tomey EM-3000 specular microscope. All images of the optic nerve head and of the fovea were acquired with the Spectralis Spectral Domain-Optical coherence tomography (SD-OCT, version 1.9.10.0 Heidelberg Engineering, Carlsbad, California, USA) by the same expert operator. The scan speed of the instrument is 40000 A-scan per second, and the scan circle diameter around the optic nerve is 12°. Typically, the scan diameter (mm) is 3.5–3.6 mm and relays on the axial eye length of the eye. The quality score of all scans is > 20 [8]. The mean RNFL thickness value and the average measurement values for all the six sectors were recorded. FT values were obtained again with the Spectralis SD-OCT, using the automated averaging system. The instrument has an axial and transverse resolution respectively less than 7 μm and 10 μm [9–10]. Automatically, the Spectralis software identifies the retinal thickness as the space between the inner limiting membrane and the complex retinal pigment epithelium-Bruch’s membrane-choriocapillaris [9]. Central foveal subfield (CSF) thickness was defined as the average of all points within the inner circle of 1-mm radius from the fovea; maximum and minimum foveal thickness values were also recorded [9]. The instrument’s camera has an internal fixation source centred on the patient’s fovea. Independently, the operator checks patient’s fixation through an infrared camera [10].

## Statistical analysis

Data were organised in a Microsoft Excel XP table to perform statistical analysis. The Kolmogorov-Smirnov test was used to assess normality of data. Pearson (r) and Spearman (rho) correlation coefficients were calculated to quantify associations between Gaussian and skewed variables, respectively. Changes at follow-up were calculated as the difference between follow-up and baseline measurements, and were analysed by paired Student’s t test when assessing differences in mean values. A p-value <0.05 was considered statistically significant. Data are presented as mean ± standard deviation (SD). Analyses were performed using SPSS (version 21, IBM Corp.)

## Results

### Results at baseline

Of the 57 patients enrolled, 46 were women and 11 were male. Their anthropometric measures are listed in Table 2 and S1 File.

**Table 2:** Anthropometric parameters and blood pressure values registered before (baseline), after 3 months and one year after bariatric surgery.

Parameter	Mean Value ±SD Baseline	Range	Mean Value ±SD After 3 months	Mean Value ±SD After 1 year
Body Weight (Kg)	117,9 ± 14,6	82.3–177,0	112,1± 24,9*	84,4± 10,8*
Height (cm)	163,7 ± 2,8	152,0–194	163,7 ± 2,8	163,7 ± 2,8
BMI (kg/m^2^)	44,1 ± 6	34,2–71,2	37,4 ± 6,4*	32,6 ± 5.5*
SBP (mmHg)	134,2 ± 20,7	100–195	124,6 ± 13,5*	123,3 ± 12,2*
DBP (mmHg)	88,6 ± 12,7	70–140	79,4 ± 7,3*	80,4 ± 7,2*

BMI (body mass index), SBP (systolic blood pressure), DBP (diastolic blood pressure), SD (standard deviation).

* paired Student’s t test, p<0.05 vs baseline.

Twenty patients were on treatment for hypertension. All ophthalmologic values registered at baseline are shown in Table 3 and S1 File. No significant differences were observed between the two eyes.

**Table 3:** Ophthalmic parameters of the right eye (RE) and the left eye (LE) before (baseline), 3 months and one year after bariatric surgery.

	Mean Value ± SD Baseline RE	Mean Value ± SD 3 Months RE	Mean Value ± SD 1 Year RE	Mean Value ± SD Baseline LE	Mean Value ± SD 3 Months LE	Mean Value ± SD 1 Year LE
VA (Snellen equivalent)	20/25 ± 20/80	20/25± 20/200	20/25 ± 20/160	20/25 ±20/200	20/25± 20/160	20/20 ± 20/200
IOP (mmHg)	15,6 ± 3,5	14,5 ± 2,5	15,9 ± 2,6	14,9 ± 3,4	14,8± 2,5	16,0± 2,6
CCT (µm)	531,1± 41,6	530,1± 39,2	531,3 ± 40.9	520,8 ± 32,0	520,1 ± 36,2	523,9 ± 31,3
FT mean value (µm)	235,4 ± 26,7	239,0 ± 28,5	239,5 ± 24,4	231,1 ± 17,5	230,3± 23,0	236,9 ± 23,9*
FT minumum value (µm)	221,4 ± 14,5	220,6 ± 13,7	224,1 ± 15,9*	221,3 ± 12,6	219,4 ± 13,2	224,0 ± 14,9*
FT maximum value (µm)	328,2 ± 21,9	326,4± 22.5	332,5 ± 25,5	327,3 ± 21,7	323,8± 19,9	331,0 ± 20,4
RNFL mean value (µm)	100,3 ± 10,4	100,1 ± 10,9	102,0± 12,5	99,0 ± 9,4	98,6 ± 9,5	99,1 ± 9.9
RNFL superior sector (µm)	113,9 ± 17,5	115,0 ± 15,7	119,2 ± 16,2*	117,4 ± 15,6	120,0 ± 15,1	124,1± 16,5*
RNFL nasal sector (µm)	78,9 ± 13,6	79,9 ± 16,6	81,2 ± 19,2	69,5 ± 10,9	77,7 ± 13,0	75,4 ± 16,5*
RNFL inferior sector (µm)	135,2 ± 18,4	132,6 ± 19,6	136,30 ± 26,1	130,8 ± 15,1	129,7 ± 15,2	129,1 ± 15,5
RNFL temporal sector (µm)	73,4 ± 13,6	72,9 ± 14,8	73,0 ± 17,2	69,5 ± 11,8	69,3 ± 12,3	70,5 ± 13,3

VA (visual acuity), IOP (intraocular pressure), CCT (central corneal thickness), FT (foveal thickness), RNFL (retinal nerve fiber layer) thickness.

* paired Student’s t test, p<0.05 vs baseline.

**Fig 1:**
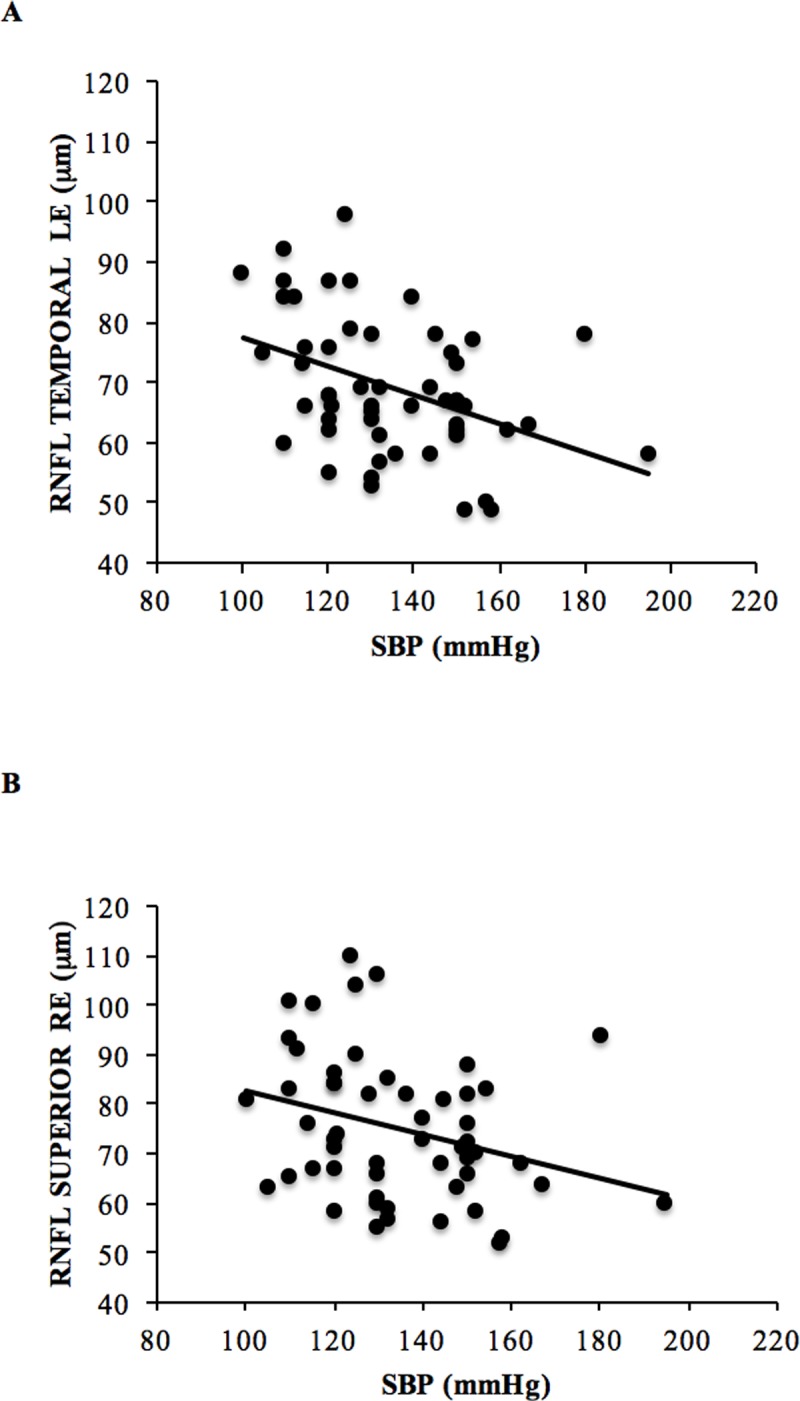
Relationship between systolic blood pressure (SBP) and retinal nerve fiber layer (RNFL) thickness. Relationship between systolic blood pressure (SBP) and retinal nerve fiber layer (RNFL) thickness before bariatric surgery in (A) the temporal sector of the left eye (LE) (R—0.43, p< 0.01) and in (B) the superior sector of the right eye (RE) (R -0,3, p<0,05).

In the univariate analysis blood pressure values were directly and significantly related to intraocular pressure values. In the right eye there was a direct association both for diastolic and systolic pressure (RE p<0.05, R = 0.35), while in the left eye only the diastolic pressure was associated with intraocular pressure values. Systolic blood pressure values remained significantly associated with IOP in both eyes, even after correction for age and body mass index, explaining 8–9% of IOP variability. Furthermore, BP values were significantly and inversely related to RNFL thickness, particularly in the temporal sector of the left eye (LE p<0.01, R = -0.43) and in the superior sector of the right eye (p<0.05, R = -0.3) (Fig 1).

BMI values were significantly and inversely related to minimum foveal thickness values in both eyes (OD p<0.02, R 0.40; OS p<0.02, R 0.30). Blood glucose, glycated hemoglobin, fasting insulin, creatinine, fasting leptin, vitamin D, vitamin B, folic acid and lipid profile did not correlate with eye parameters before surgery.

Using the LOCS III system for classification, cataract was observed in 8 patients (14%); 1 patient was classified as NO 4 C2 P1 in the right eye 3 were classified as NO 3 C1 P1; other 3 patients as NO 2 C1 P1 and 1 as NO 1 C2 and P1.

### Effects of bariatric surgery

Three months after bariatric surgery, a significant reduction of BMI value (p< 0.05), associated with a statistically significant improvement of BP values (p< 0.05) was observed (Table 2 and S1 File). The ophthalmologic examination revealed an increase of RNFL thickness in the superior sector of both eyes and an increase of FT minimum values in both eyes.

After one year, a marked weight reduction was observed. RNFL thickness increased further in the superior sector in both eyes (p<0.005), and in the left eye also in the inferior and temporal sector (p<0.05) (Table 3 and S1 File). Among 13 patients treated for hypertension, only 6 were still taking antihypertensive drugs.

**Fig 2:**
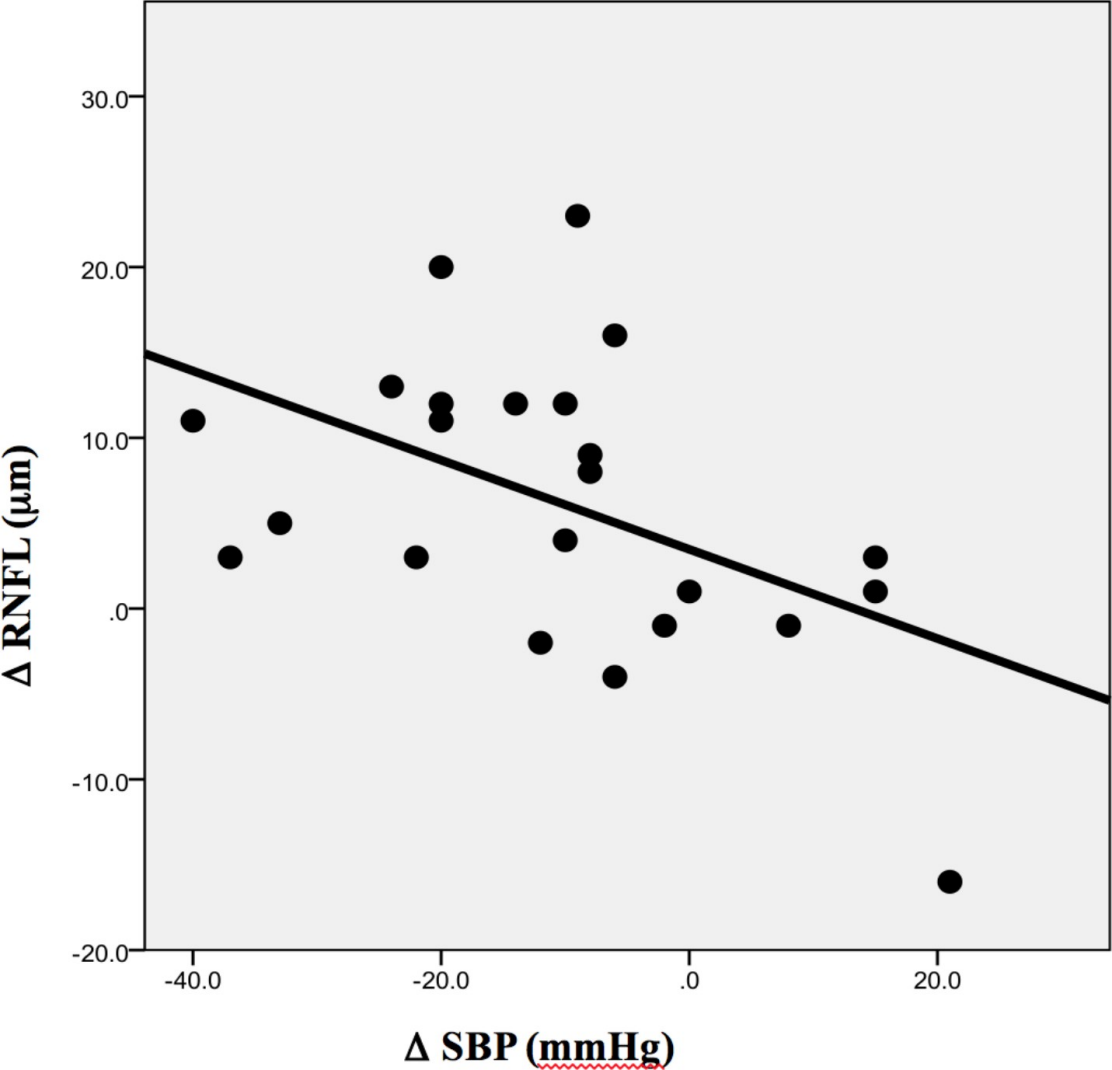
Changes of SBP (mmHg) and RNFL thickness (μm) in the superior sector of the left eye after bariatric surgery. A significant and inverse association was observed (p<0.02, R = 0.45). Data are expressed as variation (Δ) from baseline of SBP (systolic blood pressure) and RNFL (retinal nerve fiber layer) thickness.

The absolute decrease of SBP was significantly and inversely associated with an increase in the RNFL thickness in the superior sector of the left eye (p<0.02, R = 0.45), to confirm the inversely association found at baseline (Fig 2).

Additionally, there was a significant increase of minimum FT value in both eyes (RE p<0.04; LE p<0.001) (Tab 3).

Lens opacities, defined using the LOCS III classification system, did not show any change in all 29 patients three months and one year after GBP.

## Discussion

From an ophthalmologic point of view, all patients enrolled in this study can be defined as naive since they did not have a history of ophthalmologic diseases particularly glaucoma, retinopathy or AMD. Blood pressure was as an independent determinant of IOP, and was inversely related to RNFL thickness. Despite the limitation of a single BP measurement at each time point of the study, it is tempting to hypothesize that severely obese patients may have an initial, subclinical damage of the RNFL, somehow related to increased blood pressure values. Indeed, Dogan et al. [11] observed a significant association between obesity and RNFL, and changes in systemic blood pressures were found directly and significantly associated with changes in IOP and RNFL thickness [12–15].

As expected, body weight loss after bariatric surgery was remarkable. Consequent to BMI reduction, blood pressure values, both systolic and diastolic, were significantly reduced and most patients could discontinue or reduce their anti-hypertensive medications. In parallel with BP reduction, RNFL thickness increased significantly in our population, supporting the hypothesis that blood pressure may affect the RNFL. In line with this view, studies in hypertensive patients suggest that anti-hypertensive medications may be protective against glaucoma [16]. The relationship between BP and IOP could be explained by the trans-lamina cribrosa pressure difference (TLCPD), and increased TLCPD has been associated with decreased neuroretinal rim and increased visual field defects [17,18]. In our study the medical therapy for hypertension was withdrawn in all but six patients, with no apparent effect of treatment discontinuation on RNFL thickness. Unfortunately, these numbers are too limited to help answering the question whether anti-hypertensive drugs have an effect on IOP independently from blood pressure lowering.

Burgansky-Eliash [19] and Çekic [20] suggested the possibility that reduction of blood pressure after sleeve gastrectomy in obese patients may be associated with a reduction of IOP and a better retrobulbar blood flow. Increased risk for primary open-angle glaucoma (POAG) in obese subjects has been reported [21] and beside the increase of IOP, several others may be the mechanisms of damage involved: trabecular meshwork malfunctioning [22], increased oxidative stress [23], decreased aqueous outflow caused by increased orbital fat [24] and impairment of retrobulbar blood flow [20].

Yet, the actual interplay between BMI and POAG represents a debated question [25–32]. Racial variations between the studied populations may represent a possible explanation of differences among various studies. Moreover, BMI does not differentiate between fat and muscle and some studies have looked into the influence of other obesity markers on IOP such us waist circumference, waist to hip ratio, total body fat mass, and fat percentage [12, 25–29].

The second relevant observation of our study is the inverse relationship between BMI and minimum foveal thickness values. This association may give explanation for the greater susceptibility of the obese population to develop AMD. In 2001 Schaumberg et al. studied a cohort of 21,121 men participating in the Physician’s Health Study. Men with normal BMI had lowest incidence of dry AMD. The AMD incidence was not related to age and smoking and did not show any correlation with diabetes or hypertension [33]. Following reports confirmed this observation [34–38]. Since obesity is a systemic condition, the mechanisms possibly causing AMD may be various including low-grade chronic inflammation and increased oxidative damage [23], which were not investigated in the current study. After bariatric surgery, we observed a significant increase, remaining within normal limits, of minimum FT. These findings, if confirmed by larger studies, may support the role of obesity in the pathogenesis of AMD. Based on our results we speculate that the process leading to AMD might be reversible after weight loss.

Lens opacities remained unchanged after surgery. The short follow-up of the study and the young age of our population do not allow a definite conclusion. Yet, our results are reassuring about the potential adverse effects of bariatric surgery and fast weight reduction on crystalline transparency in the short run.

Limitations of our study are represented by the small sample size of the bariatric surgery group, the absence of additional control groups (e.g. lean hypertensive patients before and after pharmacological correction of blood pressure values) and the lack of anterior eye structural endpoints such as CCM or Oculus Pentacam. Furthermore, our study-population was mainly composed by women since the F:M ratio in bariatric studies enrolling consecutive patients is typically in favour of women. We acknowledge that this is a limitation and larger numbers are required to verify the potential gender effect on the studied parameters.

In conclusion, severely obese patients may be at risk of developing glaucoma and age-related macular degeneration. Body weight reduction and improvement of comorbidities achieved by bariatric surgery may be effective in preventing eye disease development by improving RNFL and foveal thickness. Optical coherence tomography, together with a complete eye examination, is a quick and non-invasive exam that may be useful in severely obese patients to detect and classify early change of the optic nerve head and the fovea, even in the absence of a history of eye diseases.

## Supporting information

S1 File Data of patients before, three months and one year after bariatric surgery. Anthropometric parameters, biochemistry, blood pressure values and ophthalmic parameters tested before (baseline), three months and one year after surgery. (https://doi.org/10.5061/dryad.1g6vm34).
(XLSX)
